# Supplementary material for: Insights into the structure and assembly of a bacterial cellulose secretion system
Source: Nat Commun. 2017 Dec 12;8:2065. doi: 10.1038/s41467-017-01523-2 (PMC5727187; doi:10.1038/s41467-017-01523-2)
Supplement: Supplementary file 1 — SupplementaryInformation [file 41467_2017_1523_MOESM1_ESM.pdf]

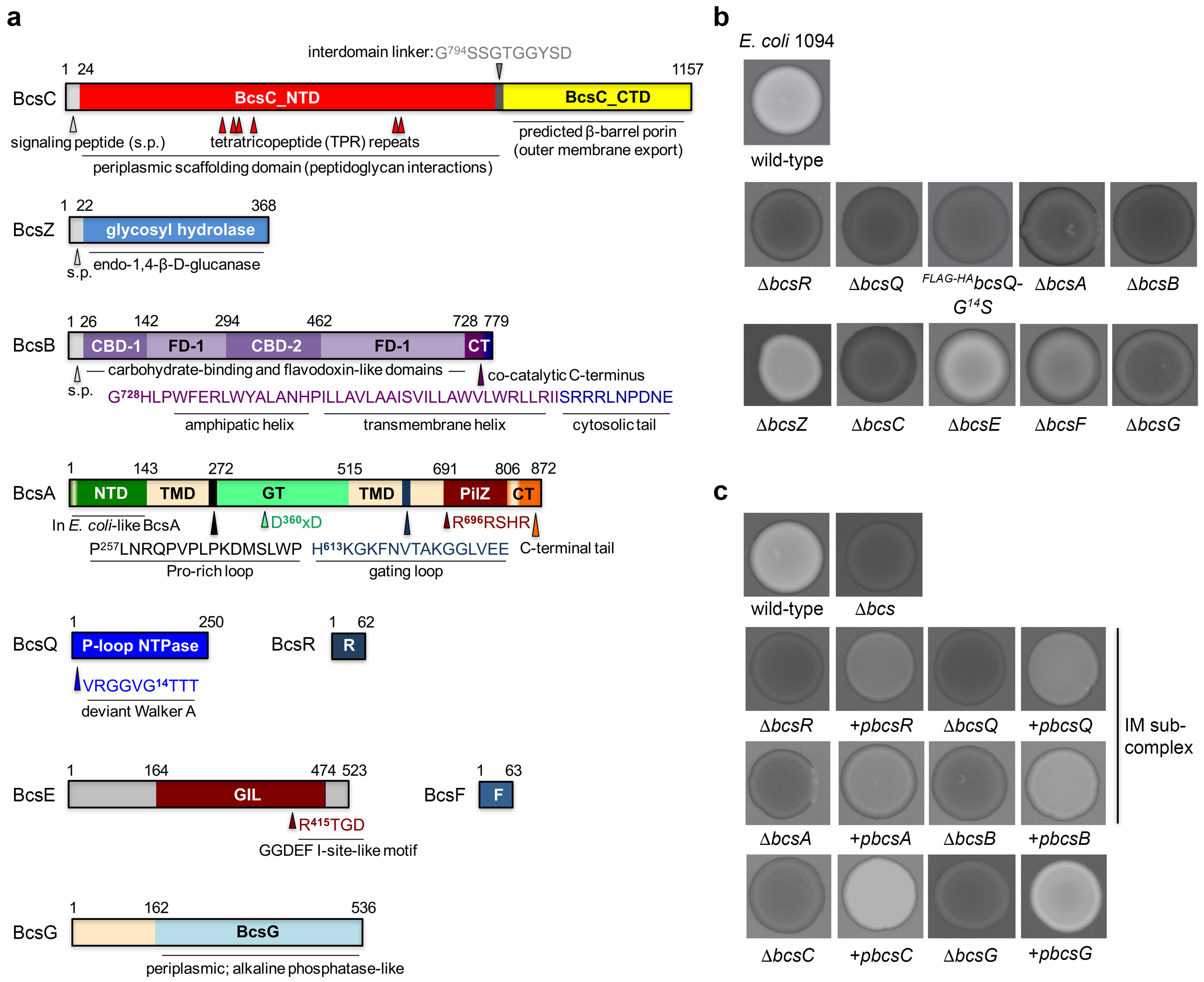

Supplementary Fig. 1: *E. coli* cellulose secretion: Bcs subunits and functional role of key subunits. **(a)** Domain organization and key structure-function motifs of individual subunits based on bioinformatics (CDD, NCBI) and structural analyses. **(b)** Cellulose secretion phenotypes of point-mutant and deletion strains as visualized by colony calcofluor binding and fluorescence. **(c)** Functional complementation of the deletion strains showing pronounced phenotypes in **b** by plasmid-driven expression of the corresponding subunits. IM subcomplex: inner membrane subcomplex (see below).

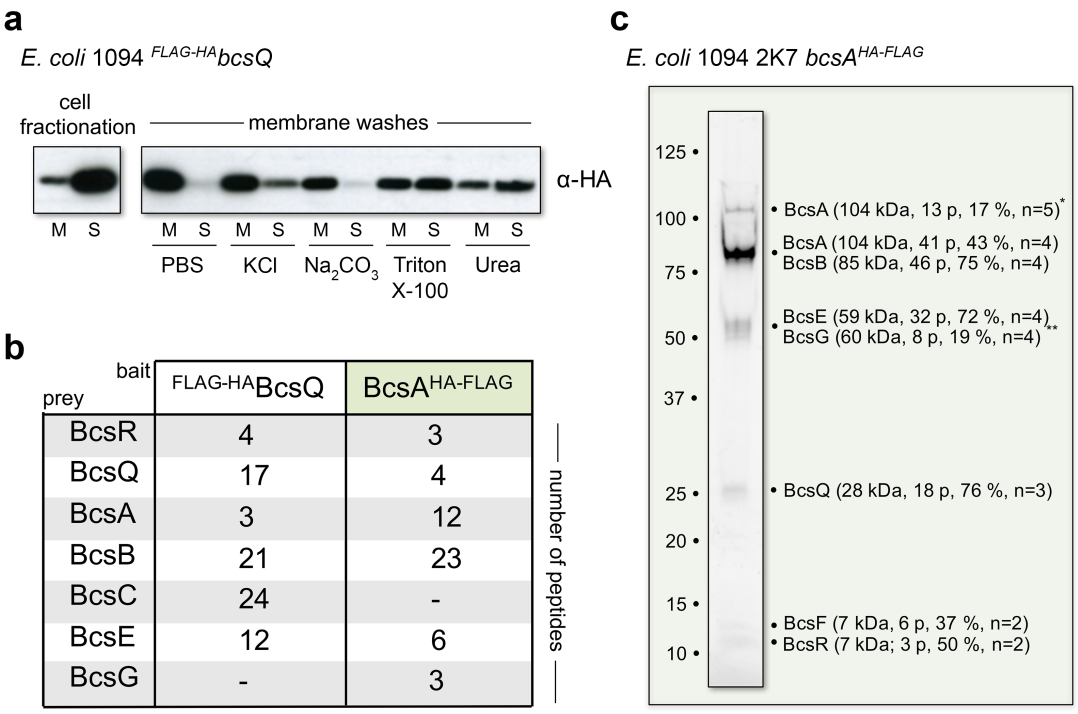

prey \ bait	FLAG-HABcsQ	BcsAHA-FLAG
BcsR	4	3
BcsQ	17	4
BcsA	3	12
BcsB	21	23
BcsC	24	-
BcsE	12	6
BcsG	-	3

(number of peptides)

Supplementary Fig. 2: Bcs macrocomplex detection. (a) Membrane association of requisite for secretion BcsQ. Cellular fractionation and membrane washing experiments on the *E. coli* 1094 $^{FLAG\text{-}HA}$*bcsQ* strain coupled with anti-HA immunoblotting. **(b)** BcsQ- and BcsA-interacting partners detected upon anti-FLAG affinity purification and mass spectrometry analyses. Detected Bcs interactions upon total elution fraction analyses on the *E. coli* 1094 $^{FLAG\text{-}HA}$*bcsQ* and *E. coli* 1094 *bcsA*$^{HA\text{-}FLAG}$ strains are shown. **(c)** Bcs protein detection in excised SDS-PAGE gel bands following *bcs* operon overexpression and affinity purification using BcsA$^{HA\text{-}FLAG}$ as bait (kDa: predicted molecular weight in kilodaltons, p: unique peptides, %: percent sequence coverage, n: number of biological replicates). Asterisks denote consistently identified contaminants as follows: *, mechanosensitive channel MscK (127 kDa) and **, ATP-synthase α-subunit AtpA and dihydrolipoyl dehydrogenase LpdA (55 and 52 kDa, respectively). All three proteins are internal membrane proteins (MscK, AtpA, and LpdA) and/or known to co-purify in FLAG tag-based membrane protein purification (LpdD, AceE and AtpA)[1,2].

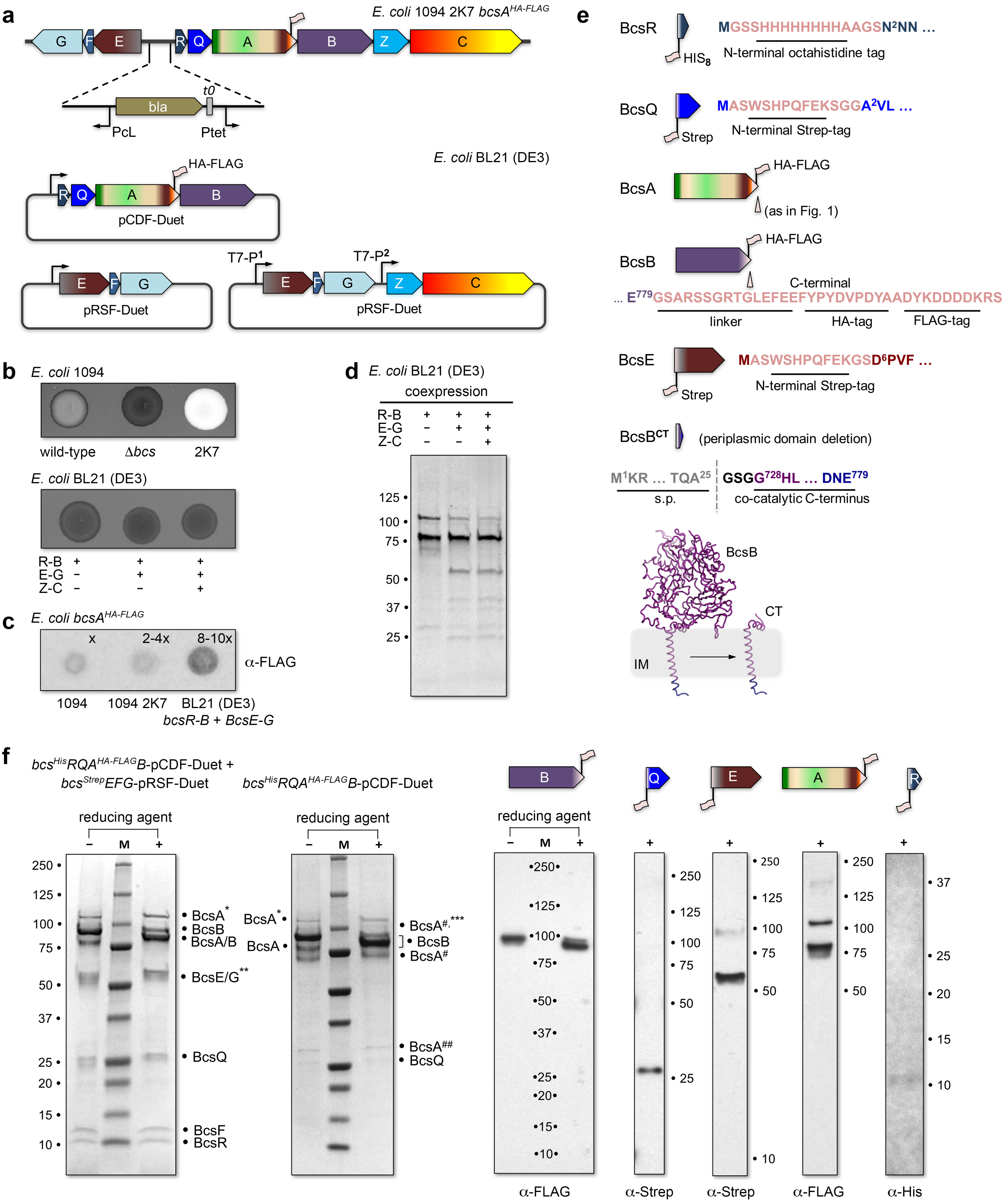

a
E. coli 1094 2K7 bcsA^HA-FLAG
G F E R Q A B Z C
bla t0
PcL Ptet
E. coli BL21 (DE3)
HA-FLAG
pCDF-Duet
pRSF-Duet
T7-P1 T7-P2
b
E. coli 1094
wild-type Δbcs 2K7
E. coli BL21 (DE3)
R-B E-G Z-C
c
E. coli bcsA^HA-FLAG
x 2-4x 8-10x
α-FLAG
1094 1094 2K7 BL21 (DE3)
bcsR-B + BcsE-G
d
E. coli BL21 (DE3)
coexpression
125 100 75 50 37 25
e
BcsR MGSSHHHHHHHHAAGSN²NN ...
HIS8 N-terminal octahistidine tag
BcsQ MASWSHPQFEKSGGA²VL ...
Strep N-terminal Strep-tag
BcsA HA-FLAG (as in Fig. 1)
BcsB HA-FLAG C-terminal
... E779GSARSSGRTGLEFEEFYPYDVPDYAADYKDDDDKRS
linker HA-tag FLAG-tag
BcsE MASWSHPQFEKGSD⁶PVF ...
Strep N-terminal Strep-tag
BcsBCT (periplasmic domain deletion)
M¹KR ... TQA25 GSGG728HL ... DNE779
s.p. co-catalytic C-terminus
BcsB CT IM
f
bcsHisRQAHA-FLAGB-pCDF-Duet +
bcsStrepEFG-pRSF-Duet
bcsHisRQAHA-FLAGB-pCDF-Duet
reducing agent
– M +
250 125 100 75 50 37 25 20 15 10
BcsA* BcsB BcsA/B BcsE/G** BcsQ BcsF BcsR
BcsA* BcsA
BcsA#,*** BcsB BcsA# BcsA## BcsQ
α-FLAG α-Strep α-Strep α-FLAG α-His

Supplementary Fig. 3: Chromosome- and vector-driven overexpression for structural studies. (a) Overexpression strategy for chromosome-driven (top) and vector-driven (bottom) overexpression in the *E. coli* 1094 $bcsA^{HA\text{-}FLAG}$ 2K7 and *E. coli* BL21 (DE3) strains, respectively. **(b)** Calcofluor-binding phenotypes of the overexpression strains. Top: wild-type refers to *E. coli* 1094 $bcsA^{HA\text{-}FLAG}$ cells with wild-type interoperon region; Δ*bcs* refers to negative control *E. coli* 1094 Δ*bcs* cells with deleted *bcs* gene cluster; 2K7 refers to the *E. coli* 1094 $bcsA^{HA\text{-}FLAG}$ 2K7 cells designed for constitutive expression as in **a**. Bottom: *E. coli* BL21 (DE3) cells transformed with the corresponding expression vectors as in **a**. **(c)** Dot-blot assay of $\mathrm{BcsA}^{\mathrm{HA\text{-}FLAG}}$ expression in the three different genetic backgrounds – *E. coli* 1094 $bcsA^{HA\text{-}FLAG}$, *E. coli* 1094 $bcsA^{HA\text{-}FLAG}$ 2K7 and vector-transformed *E. coli* BL21 (DE3) cells – normalized per gram of wet cells. The relative yields of pelleted cell weight are represented in x. Results are representative of two biological replicates **(d)** SDS-PAGE of the elution fraction of affinity purified samples after overexpression in *E. coli* BL21 (DE3) cells. **(e)** Subunit epitope tagging for orthogonal protein identification and $\mathrm{BcsB}^{\mathrm{CT}}$ truncation design. **(f)** Protein identification by mass-spectrometry and immunoblotting following SDS-PAGE on affinity- and density gradient-purified Bcs complexes. Results are representative of at least two biological replicates. Hashtags denote likely BcsA proteolytic fragments, asterisks denote additional consistently detected contaminants as follows: *, mechanosensitive channel MscK (127 kDa); **, ATP-synthase α-subunit AtpA and dihydrolipoyl dehydrogenase LpdA (55 and 52 kDa, respectively); and ***, pyruvate dehydrogenase E1 component AceE (100 kDa). All four proteins are internal membrane proteins (MscK, AtpA, and LpdA) and/or known to co-purify in FLAG tag-based membrane protein purification (LpdD, AceE and AtpA)[1,2].

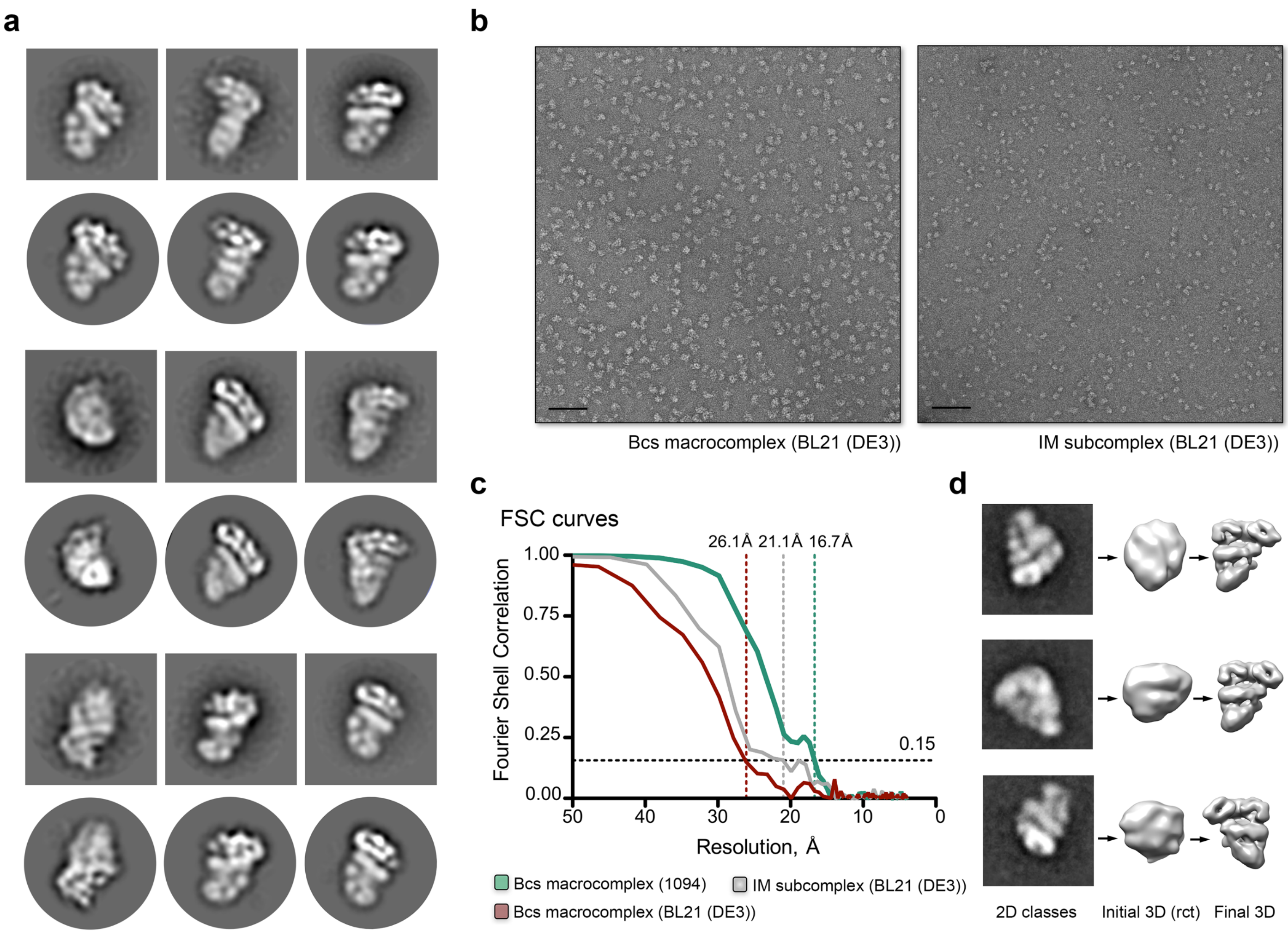

Supplementary Fig. 4: Electron microscopy data characterization. (a) Comparison between 2D class averages (squares) and 2D reprojections (circles) of the refined 3D structure reconstruction for the Bcs macrocomplex purified from *E. coli* 1094 *bcsA*$^{HA\text{-}FLAG}$ 2K7 cells. **(b)** Representative negative-stain micrographs of the Bcs macrocomplex (left) and IM subcomplex (right) expressed in BL21 (DE3) cells on the grids used for structural analyses. Scale bar, 100 nm **(c)** Fourier Shell Correlation (FSC) curves of the three Bcs complexes as calculated in Relion 1.4. **(d)** Absolute handedness ambiguity: left, untilted 2D class averages; middle, corresponding random conical tilt reconstructions obtained using 50º stage tilt and used as initial models; right, 3D models after refinement against the curated dataset used for Bcs macrocomplex structure reconstruction in Fig. 2.

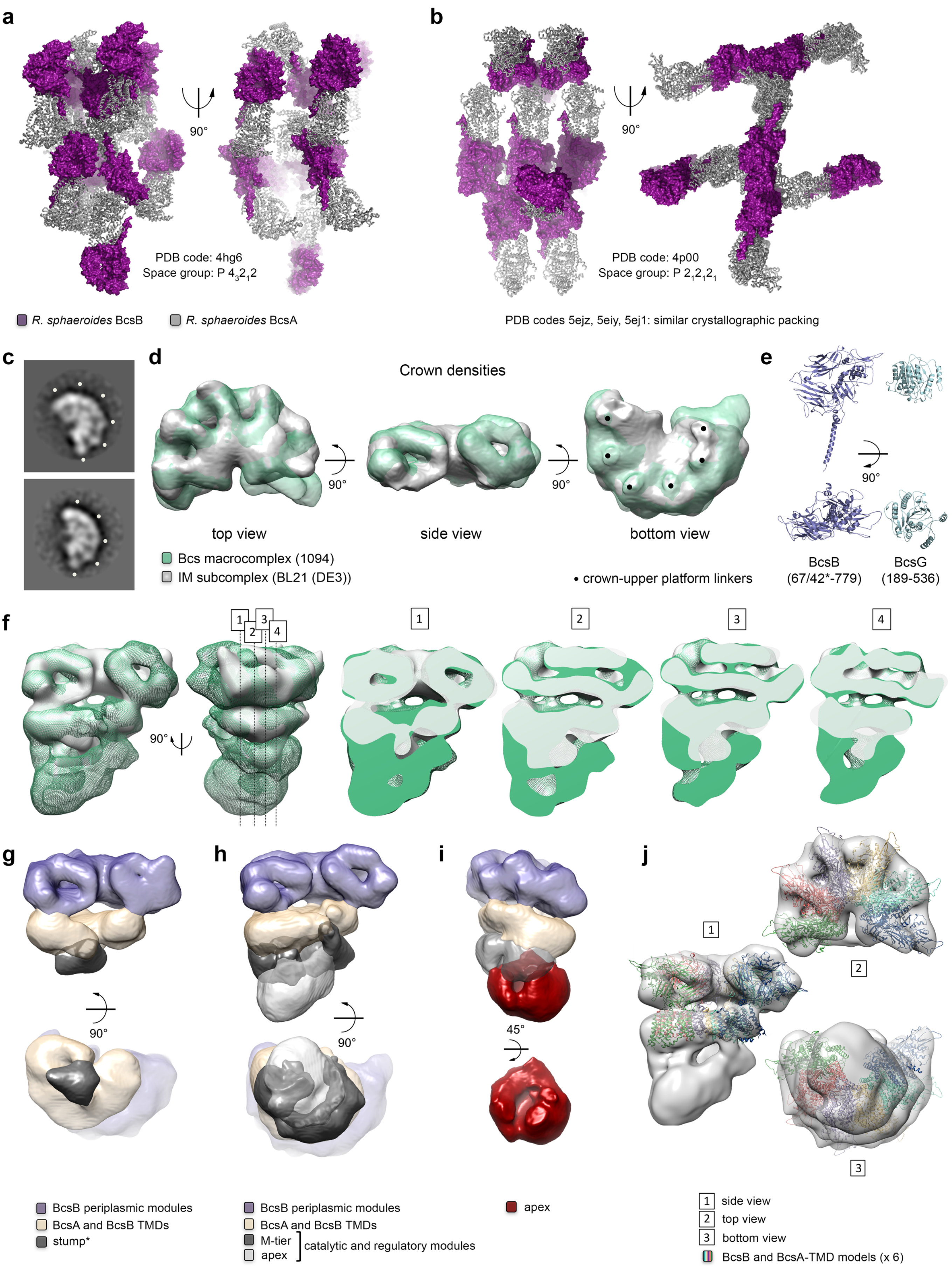

a
90°
PDB code: 4hg6
Space group: P 4_32_12
b
90°
PDB code: 4p00
Space group: P $2_12_12_1$
R. sphaeroides BcsB
R. sphaeroides BcsA
PDB codes 5ejz, 5eiy, 5ej1: similar crystallographic packing
c
d
Crown densities
90°
90°
top view
side view
bottom view
Bcs macrocomplex (1094)
IM subcomplex (BL21 (DE3))
• crown-upper platform linkers
e
90°
BcsB
(67/42*-779)
BcsG
(189-536)
f
1
2
3
4
90°
g
h
i
j
90°
90°
45°
BcsB periplasmic modules
BcsA and BcsB TMDs
stump*
BcsB periplasmic modules
BcsA and BcsB TMDs
M-tier
apex
catalytic and regulatory modules
apex
1 side view
2 top view
3 bottom view
BcsB and BcsA-TMD models (x 6)

Supplementary Fig. 5: Comparative electron microscopy analyses of the Bcs macrocomplex and IM subcomplex. **(a-b)** Crystallographic packing of the BcsAB$^{R.sphaeroides}$ duo in deposited crystal structures with antiparallel or side-by-side packing of the protomers. **(c)** Conformational heterogenity in the IM subcomplex showing prevalent representative views with 6 (top) and 5 (bottom) crown repeats. **(d)** Crown volume extraction and overlay. **(e)** BcsB and BcsG structural models generated by Robetta and shown in two different views. **(f)** Fitting of the IM subcomplex into the Bcs macrocomplex map. Colors are as in **c**. Four different cut-away views are shown on the right. **(g-i)** IM subcomplex and Bcs macrocomplex segmentation showing two different views of key structural features such as the stump (**g**), M-tier (**h**) and apex (**i**). **(j)** Fitting of structural homology models of BcsB and the transmembrane regions of BcsA into the crown and M-tier densities.

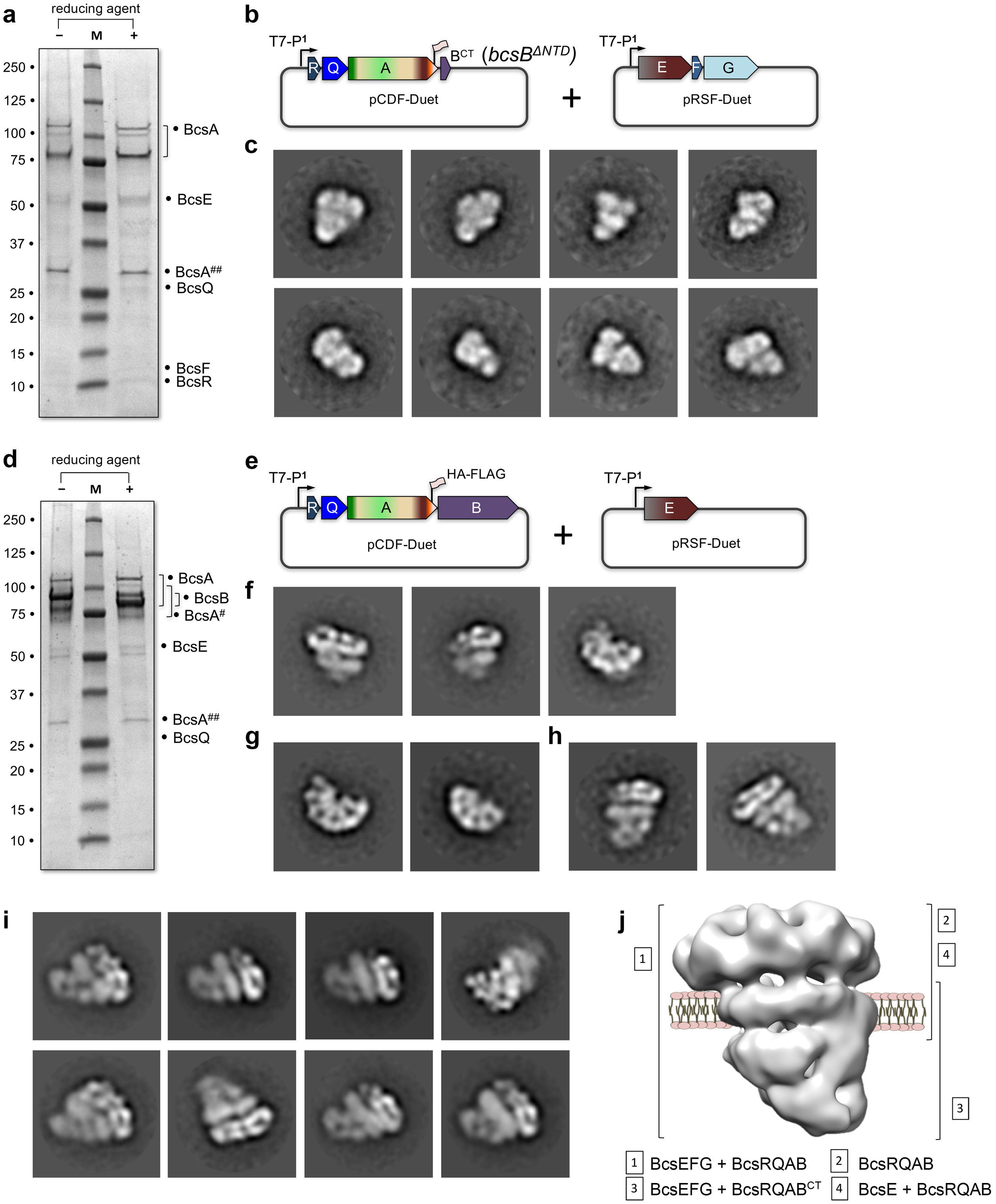

k

Expression strain / constructs	Microscope, operating voltage	Detector	Data collection	Particles for 2D class.	Particles for 3D class.	Particles for 3D reconstruction	Final resolution (FSC>0.143)
1094 *bcsA*$^{HA\text{-}FLAG}$ 2K7	Tecnai F20, 200 kV	FEI Falcon II	single tilt, NS	44 056	24 769	24 769	16.7 Å
bcs^{His}*RQA*$^{HA\text{-}FLAG}$*B + bcsEFG*	-//-	-//-	-//-	10 173	7 229	7 229	26.1 Å
bcs^{His}*RQA*$^{HA\text{-}FLAG}$*B*	-//-	-//-	-//-	127 430	20 182	14 362	21.1 Å
bcs^{His}*RQA*$^{HA\text{-}FLAG}$*B*$^{\Delta NTD}$ *+ bcsEFG*	Tecnai T12, 120 kV	Gatan Ultrascan 4000	-//-	40 845	N/A	N/A	N/A
bcs^{His}*RQA*$^{HA\text{-}FLAG}$*B + bcsE*	-//-	-//-	-//-	16 877	-//-	-//-	-//-
bcs^{His}*RQA*$^{HA\text{-}FLAG}$*B + bcs*^{Strep}*EFG*	-//-	-//-	-//-	17 193	-//-	-//-	-//-
bcs^{His}*RQA*$^{HA\text{-}FLAG}$*B + bcsEFG*	Tecnai T12, 100 kV	Gatan K2 Base	tilt pairs, 50º, NS	3633 tilt pairs	N/A	15 classes, 1 rct/class	N/A

Supplementary Fig. 6: Comparative electron microscopy analyses of Bcs deletion subcomplexes. (a) A representative coomassie-stained SDS-PAGE gel of the purified BcsRQBcsA$^{HA\text{-}FLAG}$BcsB^{CT} + BcsEFG complex (*bcsB*$^{\Delta NTD}$). **(b)** Co-expression strategy. **(c)** Representative views (2D class averages) of the purified complex. **(d)** A representative coomassie-stained SDS-PAGE gel of the purified BcsRQBcsA$^{HA\text{-}FLAG}$BcsB + BcsE complex (*ΔbcsFG*). **(e)** Co-expression strategy. **(f-h)** Representative views (class averages) of the purified complex. Views unambiguously reminiscent of the IM subcomplex (e.g. **f**, left and middle) versus the Bcs macrocomplex (**h**) are observed in 8:1 ratio. **(i)** The most populated classes of the purified BcsRQBcsA$^{HA\text{-}FLAG}$BcsB^{CT} + Bcs^{Strep}EFG complex after a single round of 2D classification. **(j)** Summary of the structural studies, showing the volume contribution and orientation of the various complexes relative to the inner membrane. **(k)** Table summarizing the EM data collection and analyses.

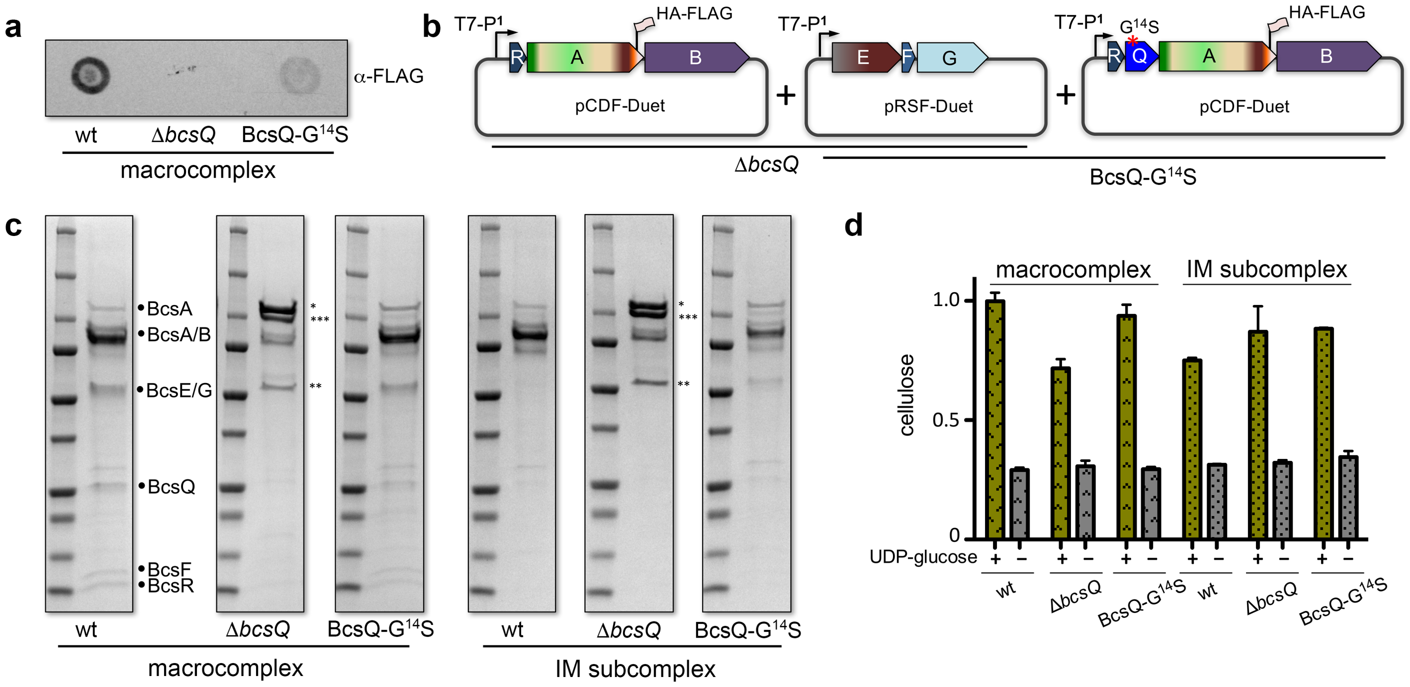

Supplementary Fig. 7: Biochemical analyses of the Δ*bcsQ* and BcsQ-G^{14}S – containing complexes. (a) Representative dot-blot assay of BcsA$^{HA\text{-}FLAG}$ expression in the two overexpression backgrounds using an anti-FLAG antibody. **(b)** Overexpression strategy used in **a**; expression cells: BL21 (DE3). **(c)** Representative SDS-PAGE gels of the three purified complexes using anti-FLAG affinity resin. Asterisks denote contaminants prevalent in the *ΔbcsQ* background: *, MscK (127 kDa); **, AtpA and LpdA (55 and 52 kDa, respectively); and ***, AceE (100kDa), as determined by mass spectrometry from two biological replicates. **(d)** *De novo* cellulose synthesis using purified complexes normalized for total protein content (two replicates).

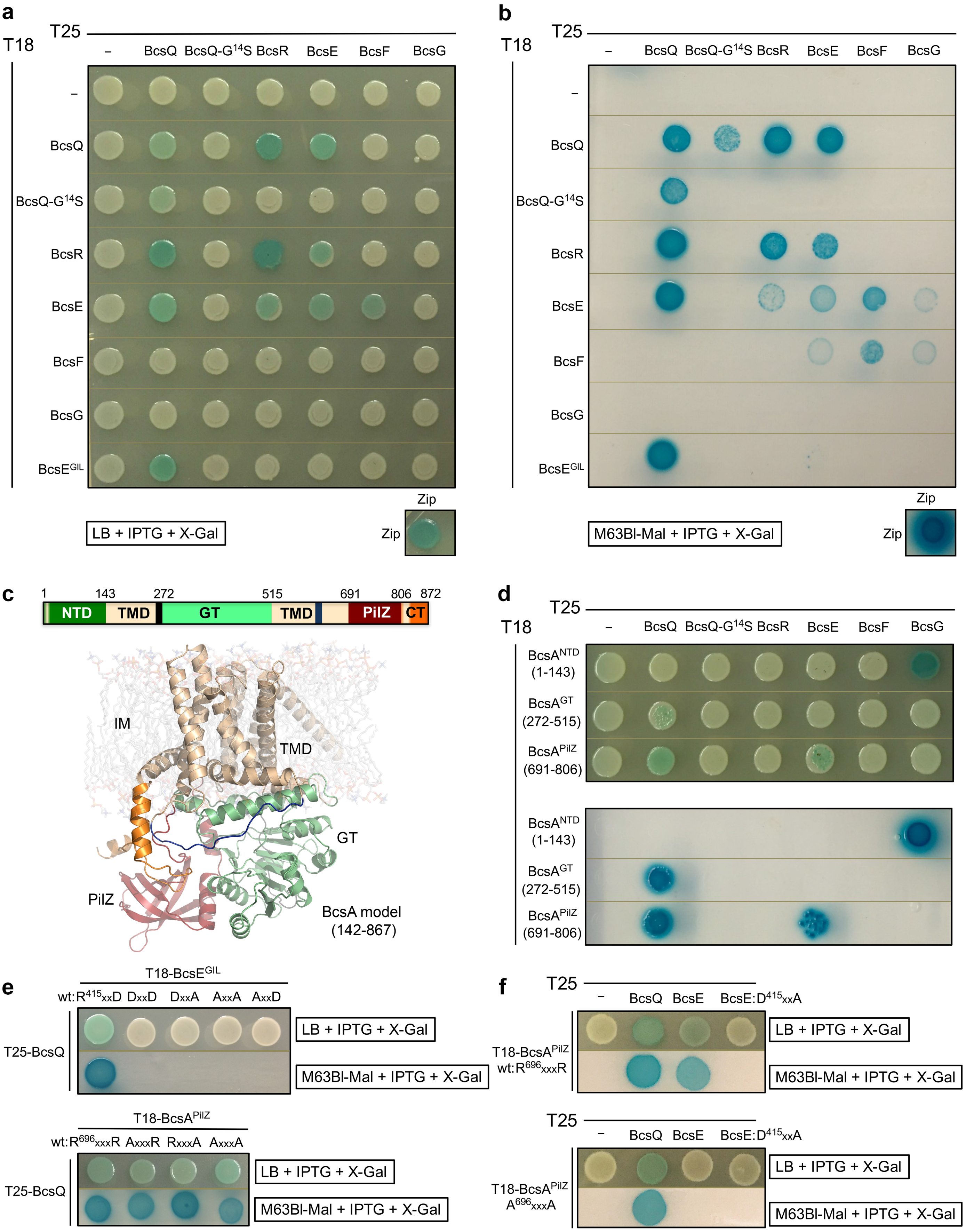
a
T25
T18
–
BcsQ
BcsQ-G14S
BcsR
BcsE
BcsF
BcsG
BcsE^GIL
Zip
LB + IPTG + X-Gal
b
M63BI-Mal + IPTG + X-Gal
c
1 143 272 515 691 806 872
NTD
TMD
GT
TMD
PilZ
CT
IM
TMD
GT
PilZ
BcsA model
(142-867)
d
BcsA^NTD (1-143)
BcsA^GT (272-515)
BcsA^PilZ (691-806)
e
T18-BcsE^GIL
wt:R415xxD DxxD DxxA AxxA AxxD
T25-BcsQ
T18-BcsA^PilZ
wt:R696xxxR AxxxR RxxxA AxxxA
f
BcsE:D415xxA
T18-BcsA^PilZ wt:R696xxxR
T18-BcsA^PilZ A696xxxA

Supplementary Fig. 8: Bacterial two-hybrid analyses of Bcs interactions. (a-b) Bacterial growth and Bcs interaction-mediated adenylate cyclase functional reconstitution (blue color) are examined on two different media with standard (LB medium, **a**) and increased (maltose-supplemented minimal medium, **b**) stringency**.** T18 and T25 denote the standard bacterial two-hybrid expression vectors encoding adenylate cyclase fragments fused to the respective Bcs subunits and subunit fragments. The positive Zip-Zip controls indicate colonies transformed with the pT25-Zip and pT18-Zip plasmids, which encode adenylate cyclase fragments fused to the homodimerizing leucine zipper regions of yeast protein GCN4. **(c)** BcsA domain organization is shown on the top; a Robetta-generated homology model based on *R. sphaeroides* BcsA is shown on the bottom. The conserved domains and structural motifs are color coded as in the domain diagram. **d.** Interactions between BcsA domains and other Bcs components as examined by bacterial growth and adenylate cyclase functional reconstitution as above. **(e-f)** Effects of c-di-GMP-binding site mutations on key binary interactions.

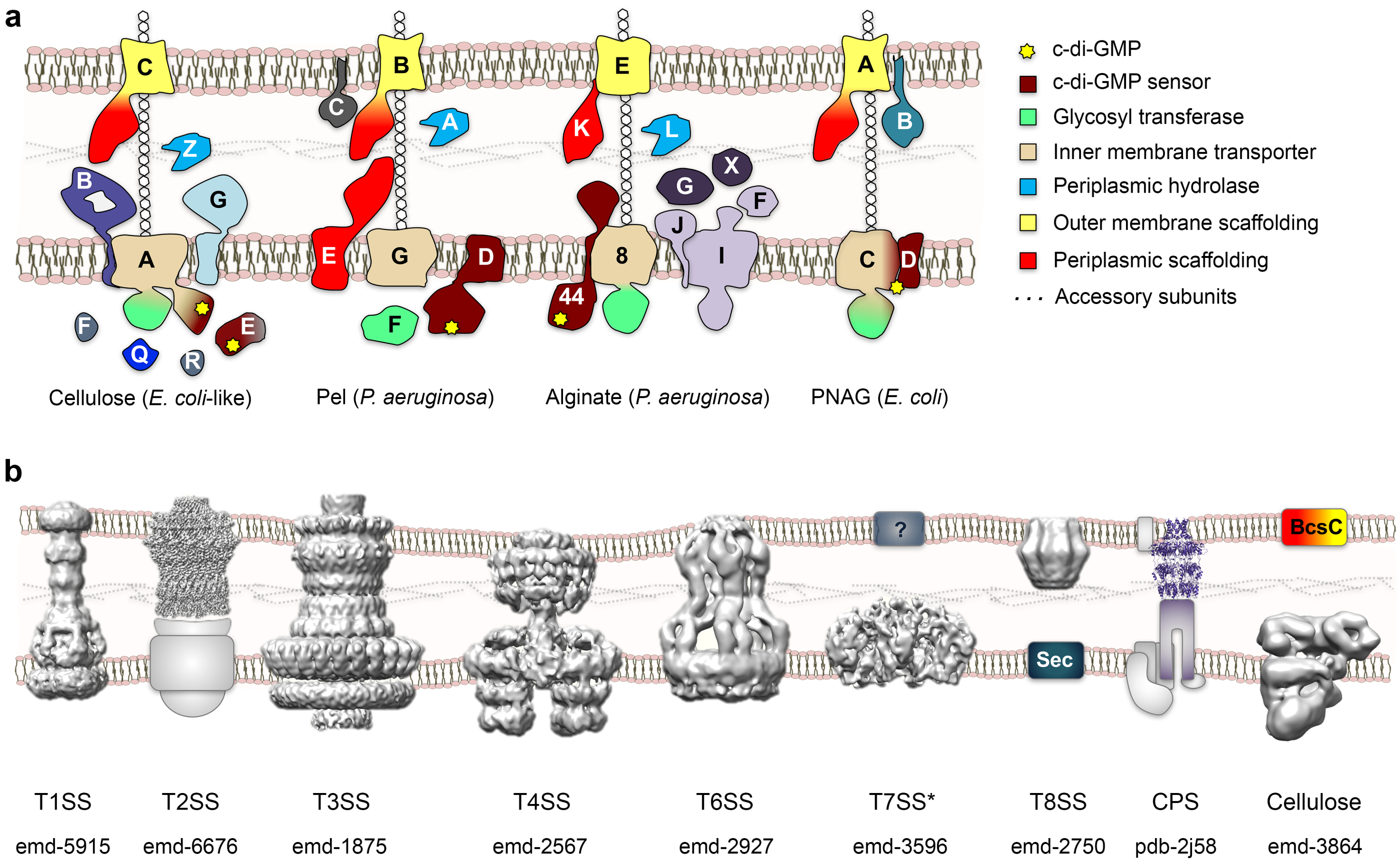

Supplementary Fig. 9: Examples of bacterial secretion systems and respective structural studies. a. Examples of synthase-dependent exopolysaccharide secretion systems in Gram-negative bacteria[3]. Functionally homologous proteins are color-coded according to the legend on the right. **b.** Structural comparison of the Bcs macrocomplex with other bacterial secretion systems, such as protein and DNA secretion systems. From left to right: the *E. coli* AcrAB-TolC multi-drug efflux pump at 15 Å resolution[4], the *Vibrio cholerae* type II secretion system secretin GspD at 3.3 Å resolution[5], the *Salmonella enterica* serovar Typhimurium type III secretion system at 11.7 Å resolution[6], the type IV secretion system of the *E. coli* R388 conjugative plasmid at 20 Å resolution[7], the membrane core complex of a type VI secretion system from enteroaggegative *E. coli* at 11.5 Å resolution[8], the *Mycobacterium xenopi* type VII secretion system membrane complex at 13 Å resolution[9], the outer membrane CsgG-CsgE nonameric complex of *E. coli* type VIII/curli secretion system at 24 Å resolution[10], the Wza outer membrane translocon for flippase-dependent type I capsular polysaccharide (CPS) synthesis in *E. coli* at 2.25 Å resolution[11,12] and the inner membrane embedded Bcs macrocomplex of *E. coli* cellulose secretion system at 16.7 Å resolution (this study). High-resolution structures of subcomplexes and individual subunits are available for most presented secretion systems but are omitted for clarity. Electron Microscopy Data Bank accession numbers are shown on the bottom. An asterisk distinguishes mycobacterial from Gram-negative systems.

References

1. Krogh, A., Larsson, B., von Heijne, G. & Sonnhammer, E.L. Predicting transmembrane protein topology with a hidden Markov model: application to complete genomes. *J Mol Biol* **305**, 567-80 (2001).
2. Li, G. & Young, K.D. Isolation and identification of new inner membrane-associated proteins that localize to cell poles in Escherichia coli. *Mol Microbiol* **84**, 276-95 (2012).
3. Krasteva, P.V. & Sondermann, H. Versatile modes of cellular regulation via cyclic dinucleotides. *Nat Chem Biol* **13**, 350-359 (2017).
4. Du, D. et al. Structure of the AcrAB-TolC multidrug efflux pump. *Nature* **509**, 512-5 (2014).
5. Yan, Z., Yin, M., Xu, D., Zhu, Y. & Li, X. Structural insights into the secretin translocation channel in the type II secretion system. *Nat Struct Mol Biol* **24**, 177-183 (2017).
6. Schraidt, O. & Marlovits, T.C. Three-dimensional model of Salmonella's needle complex at subnanometer resolution. *Science* **331**, 1192-5 (2011).
7. Low, H.H. et al. Structure of a type IV secretion system. *Nature* **508**, 550-3 (2014).
8. Durand, E. et al. Biogenesis and structure of a type VI secretion membrane core complex. *Nature* **523**, 555-60 (2015).
9. Beckham, K.S. et al. Structure of the mycobacterial ESX-5 type VII secretion system membrane complex by single-particle analysis. *Nat Microbiol* **2**, 17047 (2017).
10. Goyal, P. et al. Structural and mechanistic insights into the bacterial amyloid secretion channel CsgG. *Nature* **516**, 250-3 (2014).
11. Collins, R.F. et al. The 3D structure of a periplasm-spanning platform required for assembly of group 1 capsular polysaccharides in Escherichia coli. *Proc Natl Acad Sci U S A* **104**, 2390-5 (2007).
12. Dong, C. et al. Wza the translocon for E. coli capsular polysaccharides defines a new class of membrane protein. *Nature* **444**, 226-9 (2006).
